# Bank of Standardized Stimuli (BOSS): Dutch Names for 1400 Photographs

**DOI:** 10.5334/joc.180

**Published:** 2021-07-23

**Authors:** C. Decuyper, M. Brysbaert, M. B. Brodeur, A. S. Meyer

**Affiliations:** 1 Max Planck Institute for Psycholinguistics, Nijmegen, The Netherlands; 2 Department of Experimental Psychology, Ghent University, Gent, Belgium; 3 Douglas Mental Health University Institute, Montréal, Canada; 4 Donders Institute for Brain, Cognition and Behaviour, Radboud University, Nijmegen, The Netherlands

**Keywords:** picture naming, Dutch, name agreement, norms

## Abstract

We present written naming norms from 153 young adult Dutch speakers for 1397 photographs (the BOSS set; see Brodeur, Dionne-Dostie, Montreuil, & Lepage, 2010; Brodeur, Guérard, & Bouras, 2014). From the norming study, we report the preferred (modal) name, alternative names, name agreement, and average object agreement. In addition, the data base includes Zipf frequency, word prevalence and Age of Acquisition for the modal picture names collected. Furthermore, we describe a subset of 359 photographs with very good name agreement and a subset of 35 photos with two common names. These sets may be particularly valuable for designing experiments. Though the participants typed the object names, comparisons with other datasets indicate that the collected norms are valuable for spoken naming studies as well.

An important challenge for psycholinguistic and neurobiological studies of speaking is how to elicit specific utterances, for instance the noun “apple”. A commonly used task is picture naming, which is seen as well-suited for studying the core processes of speech planning, including the selection of a concept, the retrieval of the corresponding word representations, and articulatory planning processes (e.g., Levelt, Roelofs, & Meyer, 1999). Numerous studies have been dedicated to unravelling the processes involved in picture naming and their neurological bases (Miozzo, Pulvermüller, & Hauk, 2015; Piai, Roelofs, Rommers, & Maris, 2015).

For any naming experiment, it is crucial to select appropriate stimuli. Items can be selected on the basis of experiment-specific pretests or/and by reference to published norms. In general, it is good to have several item sets to select from, as this allows researchers to measure processes that generalize beyond the specific stimulus set used, a requirement known in test psychology as the multimethod approach (Campbell & Fiske, 1959; Eid & Diener, 2006). In the present paper, we describe the results of a photo norming study carried out with adult native speakers of Dutch. We are convinced that the norms will be of use for researchers working with Dutch speakers, because the available normed stimulus sets consist of drawings only and are smaller in size.

Participants saw a series of photographs and typed the names. Our norming variable of main interest was name agreement (NA), which is the proportion of speakers who use the same name to refer to an image. Previous studies have shown that name agreement is a strong predictor of naming latency. Images with high name agreement are named faster and more accurately than images with low name agreement (Alario et al., 2004; Barry, Morrison, & Ellis, 1997; Bonin, Chalard, Méot, & Fayol, 2002; Bonin, Peereman, Malardier, Méot, & Chalard, 2003; Cheng, Schafer, & Akyürek, 2010; Cuetos, Ellis, & Alvarez, 1999; Dell’acqua, Lotto, & Job, 2000; Ellis & Morrison, 1998; Lachman, Shaffer, & Hennrikus, 1974; Paivio, Clark, Digdon, & Bons, 1989; Snodgrass & Yuditsky, 1996; Vitkovitch & Tyrrell, 1995).

A common interpretation of this finding is that images with low name agreement activate multiple competing conceptual and/or lexical representations, and the need to select among them slows down naming and makes it more error-prone compared to naming images with high name agreement (Alario et al., 2004; Levelt et al., 1991). Name agreement tends to affect naming speed and accuracy independently of word frequency and age of acquisition (Lachman et al., 1974; Vitkovitch & Tyrrell, 1995).

Controlling name agreement is relevant for the design of many studies. For instance, researchers may want to generate two or more sets of images differing on one feature and matched on other variables likely to affect naming performance. Name agreement should then be taken into account because of its known effect on naming speed and accuracy. For other studies researchers may want to select images that are highly likely to be named in the same way by all adult native speakers of a language. This can be classic chronometric or neurobiological studies of speech planning (e.g., Jongman, Roelofs, & Lewis, 2020), studies into naming disorders (e.g., Bose & Schafer, 2017), second language testing (Gollan, Weissberger, Runnqvist, Montoya, & Cera, 2012) or studies of verbal or visual memory (e.g., Zormpa, Brehm, Hoedemaker, & Meyer, 2019). For other studies, images may be needed that elicit two or more plausible names, such as ‘sofa’, ‘couch’ and ‘settee’ (see e.g., Jescheniak & Schriefers, 1998; Peterson & Savoy, 1998). This holds, for instance, for studies of executive control of picture naming, where naming agreement can be varied to induce more or less competition between lexical items, which needs to be resolved through the recruitment of selective inhibition (e.g., Shao, Roelofs, Acheson, & Meyer, 2014). It also holds for studies of referential communication, where speakers may or may not converge on common names (e.g., Brown-Schmidt, 2009), and for studies interested in the origins of name agreement effects in their own right (Madden, Sale, & Robinson, 2019; Vitkovitch & Tyrrell, 1995). Finally, authors may be interested in pictures that have a low name agreement, because participants may be less likely to encode such pictures verbally (e.g., Nakabayashi, Burton, Brandimonte, & Lloyd-Jones, 2012).

To select suitable images, researchers can either run pilot and norming studies or make use of existing norms (for an extensive overview of image norming studies in different languages and samples, see Brodeur, Guérard, & Bouras, 2014). Many picture naming studies have used the norms prepared by Snodgrass and Vanderwart (1980). However, the normed picture set is not large (260 pictures) and consists of black-and-white drawings, some of which are now somewhat dated. Moreover, line drawings are not optimal for all research purposes. As Brodeur et al. (2010, 2014) pointed out, black-and-white line drawings are often harder to recognize than coloured images or photographs as they miss cues related to colour and texture (see also Biederman & Ju, 1988; Brodie, Wallace, & Sharrat, 1991; Moreno-Martínez & Montoro, 2012; Ostergaard & Davidoff, 1985).

Brodeur and colleagues (2010, 2014) generated a large set of photographs, known as BOSS (Bank of Standardized Stimuli) and made them publicly available along with written naming norms collected for Canadian English. A subset of the photographs was also normed for Canadian French (Brodeur et al., 2012) and Thai (Clarke & Ludington, 2018). As in the study by Snodgrass and Vanderwart (1980), pictures were normed for *name, object familiarity* (concept rather than picture), *visual complexity* (in terms of the quantity of details and the intricacy of the lines), and *image agreement*. In addition, Brodeur and colleagues collected norms for *category* (participants could make a selection from 18 categories, such as clothing, food, furniture or “other”), *manipulability* (whether or not it is easy to mime the action associated with this object), *object agreement* (reflecting how similar the object is to the one imagine by the participant) and *viewpoint agreement* (whether or not the object is in the same position as the participant imagined the object to be in). Participants were also asked to classify objects as *living or non-living* things.

In the present paper, we describe a norming study of these photographs for speakers of Dutch. We only know of one similar study, with a much smaller set of photographs (327 items; Shao & Stiegert, 2016). We presented 1397 out of the 1468 photographs prepared and described by Brodeur and colleagues (2010, 2014) to young adult speakers of Dutch. As in the original norming studies, we asked the participants to type the names of the objects and, when they could not do so, to indicate whether they did not know the object (DKO) or did not know its name (DKN). These two categories are important as they provide clues to the reasons for naming difficulties. We did not include the category ‘tip-of-the-tongue’ (TOT) of the original study, as TOT states are unlikely to occur for the relatively high-frequent names we expected to be produced and difficult to interpret without follow-up questions. In addition to written names, we obtained ratings of object agreement by asking the participants to indicate on a 5-point scale how well each photograph represented the actual concept. To limit the duration of the study, we did not collect norms for manipulability, object viewpoint, object familiarity, and visual complexity and did not ask participants to categorize stimuli, as had been done in the studies by Brodeur and colleagues. These variables were of lesser interest to our work than NA and can, of course, be obtained from the original studies (or collected in future studies). We provide summary statistics for the items below, as well as correlational analyses, including split-half correlations as a measure of reliability, and we briefly discuss the morphological and semantic relations between competitors for items with multiple plausible names.

As the participants typed the object names, the norms are particularly suitable for studies of written language production (Bonin, Méot, Laroche, Bugaiska, & Perret, 2019; Torrance et al., 2018). However, they can also assist researchers in the generation of materials for spoken language studies. For instance, if a photograph elicits only a single written name (e.g.“apple”) it would be highly surprising to see participants use many different names in oral naming. Likewise, if a photograph elicits many different names and/or many DKO and DKN responses, it would be surprising to see participants converge on a single name in oral naming.

## Method

### Participants

All 153 paid participants were recruited from the MPI participant database (mean age = 23 years; 30 males). The composition of the sample matched the composition of the participant pool. All participants were university students, native speakers of Dutch, and reported having normal sight. None of them was color-blind. Ethical approval to conduct the study was given by the Ethics Board of the Social Sciences Faculty of Radboud University.

### Materials and design

The Canadian English version of BOSS consists of two sets of photographs (see Brodeur et al. 2010, 2014, for details on how the photographs were created). One set of 538 items was described in Brodeur et al. (2010) and a second set of 930 photographs in Brodeur et al. (2014). We tested most of these photographs, divided into three sets. Set 1 consisted of the 467 “useful” photographs in the set of 538 photographs tested by Brodeur et al. (2010). The remaining photographs were discarded as suboptimal by Brodeur et al. (2010) or O’Sullivan, Lepage, Bouras, Montreuil, and Brodeur (2012). The set of items covers a broad range of object categories, including animals; body parts; building infrastructure; building materials; clothing; decoration and gift accessories; electronic devices and accessories; food; furniture; games, toys, and entertainment; hand labour tools and accessories; household articles and cleaners; jewels and money; kitchen utensils; medical instruments and accessories; musical instruments; natural elements and vegetation; outdoor activity and sport items; skin care and bathroom items; stationary and school supplies; vehicles; weapons and items related to war.

To create sets 2 and 3, the 930 items from Brodeur et al. (2014) were randomized and split into two sets of 465 items each. The photographs were sized to 400 by 400 pixels (10.58 cm on the screen), corresponding to approximately 10 degrees of visual angle at a distance of 60 cm. Each list was seen by 50 to 52 participants. Two of the 153 participants were accidentally invited twice to participate in the study and named two sets. For each participant, a new random sequence of items was created.

### Procedure

Participants were tested individually in a quiet room. They were asked to type the names of a set of photographs and rate on a 5-point scale how well each photograph depicted the object. Photographs were presented one by one on a computer screen, together with the scale. Participants typed their response underneath the photograph and pressed Enter to end the keyboard input. They used the digits 1 to 5 (top of the keyboard) to indicate how well the photograph represented the object (“Indicate on a scale from 1 to 5 how well the photograph depicts the object”, 1 = very poor representation; 5 = perfect representation). The selected number was highlighted on the scale.

When a participant could not name an object, they were asked to press Enter to skip the keyboard input and then press “a” or “b” to indicate that they did not recognize the object (button a, “DKO”) or that they recognized the object but did not know its name (button b, “DKN”). The instructions were available to the participants on screen on every trial. The manual response (pressing 1-5 or a-b) triggered the next trial with a delay of 500 ms. Participants could not go back and change their responses. The study was run on desktop computers and laptops making use of Presentation software (NeuroBehavioral Systems Inc., 2017).

### Data coding and norms

We first identified DKN and DKO responses and excluded them from further analyses. In some trials (141 responses, i.e. only 0.20% of the data), participants did not name the picture, but did press 1-5 instead of selecting “a” or “b”. These responses were manually coded as “c” and excluded as well (but not labelled as DKN or DKO). To prepare the name agreement norms, obvious spelling errors and typos were corrected to allow for aggregation of responses. Where multiple spellings were possible (e.g. “giraf” and “giraffe”), the more common spelling was selected. Furthermore, we aggregated across all responses pertaining to the same lemma, i.e. across singular and plural forms (e.g. “amandel” and “amandelen”), and simple forms and diminutives (“hoed” and “hoedje”). Object names including adjectives counted as separate responses, as long as they defined specific types of objects (e.g. “plastic beker” (plastic cup). Other adjectives (as in “blauwe beker” (blue cup)) were ignored.

We computed Modal Name Agreement (NA) as the percentage of participants who gave the most common name. We also computed the H-value for name agreement, defined as:

H = \mathop \sum \limits_{i = 1}^k {P_i}{\log _2}\left({\frac{1}{{{P_i}}}} \right)

The H-value is sensitive to the number of different names (k) that were given to an object and the proportion of participants that used each of these names (P_i_) after excluding DKN and DKO responses. This statistic was also used by Snodgrass and Vanderwart (1980) and Brodeur et al. (2010; 2014). An object with a unique name (naming agreement = 100%), has an H-value of 0. H increases with the number of alternative names given. For Object Agreement we report the mean across participants.

To facilitate the use of the norms, we added indices of Word Frequency, Word Prevalence (WP), and Age of Acquisition (AoA) from other sources to our dataset. We report the frequency per million words (SUBTLEXWF) and its log (Lg10WF) from the commonly used SUBTLEX-NL database (Keuleers, Brysbaert, & New, 2010), as well as Zipf values on a 7-point logarithmic scale, calculated as log10(fpmw*1000) (see Van Heuven, Mandera, Keuleers, & Brysbaert, 2014 for more info; *https://osf.io/3d8cx/wiki/home/*). Word Prevalence scores, i.e. the percentage of a population knowing a word, were taken from a large online study by Keuleers, Stevens, Mandera, and Brysbaert (2015). Norms for Age of Acquisition, referring to the age at which a word was acquired, were collected and aggregated with data from a study by Brysbaert, Stevens, De Deyne, Voorspoels, and Storms (2014).

## Results and Discussion

Due to a technical error, only 465 trials were presented in all sessions (instead of 467 trials for sessions with set 1 and 465 for sets 2 and 3). However, because the items were randomized differently for each participant, two different photographs from set 1 were omitted from each participant’s list. For each photograph, a maximum of 52 responses could be obtained. Across all items, the percentages of DKO and DKN responses were 5% and 8%, respectively. For 31 out of 1397 items, fewer than 70% of participants produced a name, either because they did not know the object or because they did not know its name.

Norms for all 1397 photographs are provided in OSF Table B. This table includes the modal Dutch name for each item (modal_Name; name that was used by most participants, including inaccurate names), followed by the English (file)name (English_Name) as per Brodeur et al. (2010, 2014), the number of participants that saw this photograph (Nparts), and the number of DKO and DKN responses for this item. To calculate modal name agreement (modal_NA) and H-value, DKO and DKN (and “c”) responses were excluded first, but incorrect names were included. We also list modal name agreement as a proportion of the preferred name out of all responses, including DKO and DKN (and “c”) responses (modal_NA_all). Modal NA can be high because of one popular name or because a lot of participants did not respond, but there was consensus among those who did. (This is also helpful in the comparison with Table A2). Average object agreement (OA) was calculated over OA scores of participants using the modal name. Word Frequency (SUBTLEXWF, lg10WF, Zipf), Word Prevalence (WP) and Age of Acquisition (AoA) were added for the modal name of each item (Keuleers et al., 2010; Van Heuven et al., 2014; Brysbaert et al. 2014).

As we were also interested in alternatives for the modal names, the number of unique names per item (Names) and the two most frequent competitors (Alt1 and Alt2) to the modal name are reported, together with their Naming Agreement scores (Alt1_NA and Alt2_NA). The last two columns in the table indicate whether the modal name was a correct label for the photograph (modal_Valid; correct = 1; data were coded by multiple trained native speakers; hyper- and hyponyms, diminutives, plural-singular forms and non-standard names for the object were categorised as correct as well) and whether this item was included in the set of photographs used for further analysis (selected = 1; see below for exclusion criteria). As in Brodeur et al. (2010), photographs that were not recognized by many participants (DKO score over 20%), photographs that were named incorrectly by the majority of participants (invalid modal name), and photographs for which the most common name was used by fewer than 20% of the participants, were removed from the set of useful stimuli and put in a separate file of difficult to name pictures. In total, 208 photographs (15%) were removed from the analyses reported below.

**Figure 1:**
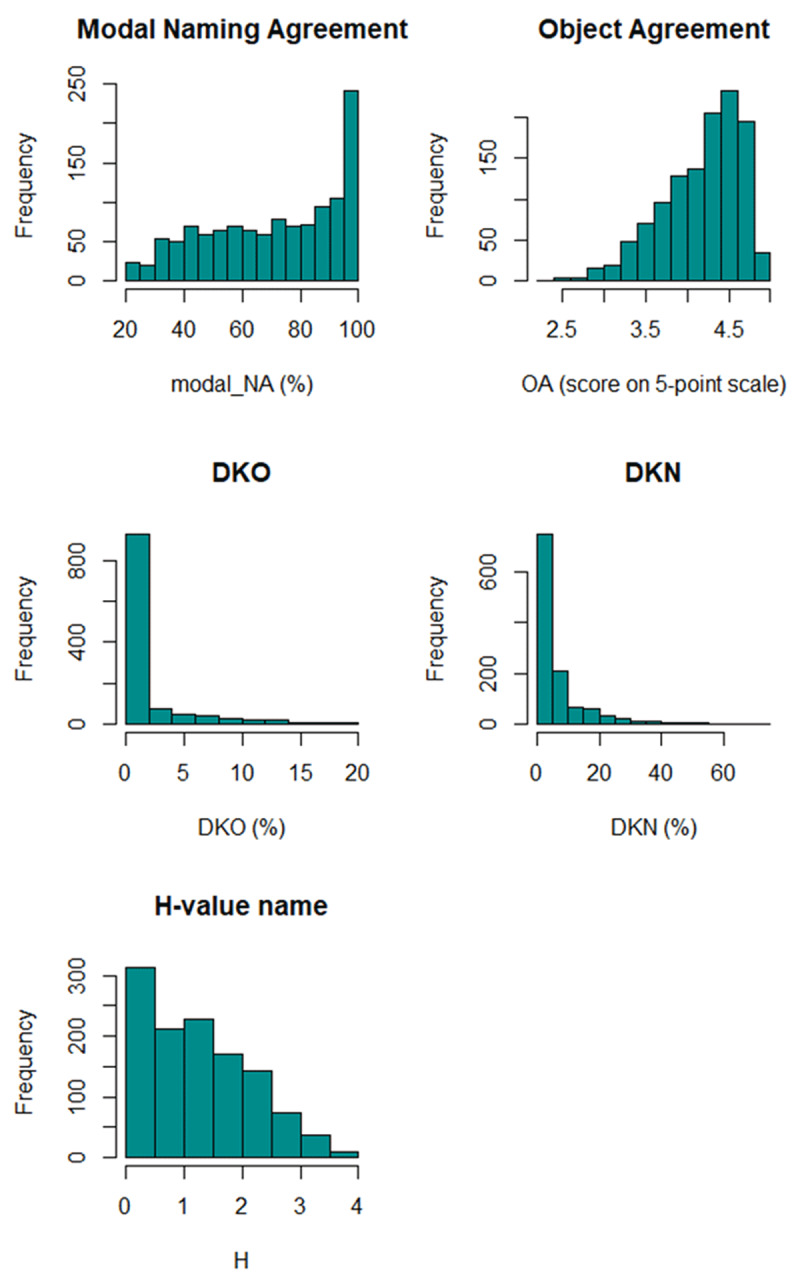
Frequency distributions of the five dependent variables in the current study.

Further analysis was carried out in R (R Core Team, 2018), with the remaining 1189 photographs (OSF TABLE C; note that invalid alternative names were excluded for Table C as well; modal NA per item is the same in Tables B and C). ***Table 1*** summarizes the norms for this set of items; ***Figure 1*** shows the frequency distribution of the norms.

**Table 1:** **Norms of the 1189 photographs**. Modal name agreement is the percentage of people that gave the most common name (calculated over the modal_NA, not modal_NA_all column in OSF Table C). H is a measure of entropy describing name agreement. DKO refers to the percentage of participant who indicated they did not recognize the object. DKN is the percentage of participant who indicated they did not know the name of the object. Object agreement is how well the photographs resembled the object shown (rated on a 5-point scale). Zipf scores for word frequency, word prevalence, and Age of Acquisition were added from different databases.


VARIABLE	MEAN	SD	MIN	MAX

Modal name agreement (%)	71	23	21	100

H-value name	1.2	0.9	0	3.7

DKO (%)	2	4	0	20

DKN (%)	6	10	0	72

Object agreement	4.2	0.5	2	5

Frequency (Zipf)	3.5	0.9	1.7	6.6

Prevalence	1.84	0.13	0.88	1.96

Age of acquistion	7	2	4	14


As can be seen in ***Table 1***, on average 2% of the participants indicated they did not know the object (sd = 4), and 6% did not know the name of the object (sd = 10). Modal naming agreement for the remaining items was 71% (sd = 23), and the average H-value for naming agreement was 1.2 (sd = 0.9). The average object agreement score was high (4.2, sd = 0.5, on a 5-point scale), showing that the photographs originally selected for Canadian participants are suitable for use in the Netherlands. The object names had an average Zipf score (word frequency) of 3.5 (sd = 0.9) corresponding to 3.16/million word. The average word prevalence was 1.84 (sd = 0.13), meaning that on average, the words were known by 97 % of the population. The AoA was 7 (sd = 2). All in all, the item set can characterized as an easy set for Dutch university students.

To determine the reliability of our norms, we computed split-half correlations. We split the dataset into two sets based on participant number (even or odd). Valid items were selected for each groups in the same way as for the entire dataset (modal name should be a correct name; DKO < 20%; modal_NA > 20%). The *even* and *odd* set each contained 1175 items. Because of the per-group filtering 90 photographs occurred in only one of the sets. Hence 1130 items were included in the correlations. We found high positive correlations for modal Name Agreement (r_s_ = 0.89, p < .001) and Object Agreement (*r_s_* = 0.79, p < .001). We also compared the dominant names for each item across groups. This was the same for 1038 out of the 1130 items (92%). All in all, the NA norms show good reliability.

***Table 2*** shows the Spearman correlations among the variables assessed in the study and garnered from other sources. As expected, we found a strong negative correlation (*r_s_* = –0.97, p <. 001) between NA and H-value, as lower H-values imply stronger agreement. Thus we only comment on the correlations of the remaining variables with NA. Name agreement correlated significantly with object agreement, showing that items rated as better representations of objects (high score on the 5-point scale) were named more consistently (higher NA) than items rated as poorer representations. As expected, name agreement correlated negatively with the proportions of DKO and DKN responses. This indicates that some objects were harder to recognize or name than others, yielding more varied and more missing responses. NA did not correlate with Zipf frequency, word prevalence, or AoA. Thus, participants were not more, or less, likely to agree on “easy” than on “harder” names. Finally, there were the well-documented significant correlations between AoA, Zipf frequency and word prevalence.

**Table 2:** Overview of Spearman’s rank correlation coefficients (*r_s_*, rho) for correlations between all variables.


	NA	H	OA	%DKO	%DKN	ZIPF	WP	AOA

NA								

H	–0.97**							

OA	0.40**	–0.45**						

%DKO	–0.24**	0.28**	–0.51**					

%DKN	–0.42**	0.49**	–0.44**	0.47**				

Zipf	0.04 *p = .20*	–0.03 *p = .25*	–0.22**	–0.05 *p = .14*	–0.12**			

WP	0.08 *p = .02*	–0.08 *p = .01*	–0.08 *p = .02*	–0.05 *p = .12*	–0.12**	0.24**		

AoA	–0.08 *p = .01*	0.09 *p =.006*	0.13**	0.11**	0.21**	–0.63**	–0.30**	


* Bonferroni correction: 28 pairwise comparisons, so significant at p-value smaller than .002 (.05/28). ** p < .001. NA = Name Agreement; H = H-value; OA = Object Agreement; %DKO = percentage of trials in which participants did not recognize the object; %DKN = percentage of trials in which participants did not know the name of the object; Zipf = Zipf score for word frequency; WP = Word Prevalence (z-scores); AoA = Age of Acquisition.

As mentioned in the Introduction, researchers often need to select items with high name agreement. To facilitate such a selection, we categorized the items in OSF Table C as “good” (NA above 90%), “fairly good” (NA between 89 and 75%) and “poor” (the remaining items). There were 359 “good” and 226 “fairly good” items. For both categories the proportions of DKO and DKN responses were low (below 6%).

Since some studies might require sets of photographs with multiple plausible names, we determined how many items there were with two frequently given names. Note that we used the raw data (checked for spelling, but not aggregated for the same lemma, see OSF Table D) for all 1397 photographs in the analyses that are described next (e.g. *vogelhuis* and *vogelhuisje* are two strong names for the item birdhouse). We found a set of 35 *strong* pairs, where each of two names was used by between 40 and 60% of the participants, and a set of 187 *plausible* pairs, where each of two names was used by 25 to 50% of the participants. (Note that there is some overlap between these sets. There are 204 unique strong/plausible pairs; See OSF Tables D1 and D2. Of course researchers can select different types of pairs on the basis of the data given in OSF Table D, or Table B if one would like to look at aggregated names).

We were interested in the nature of the pairs in the *strong* and *plausible* sets. We established whether or not the two names were morphologically related, i.e. had at least one morpheme in common, and conducted a broad assessment of semantic relatedness. We found that 48% of the pairs (97 out of 204 pairs) had morphologically unrelated names (e.g. kopje-mok; cup-mug). These items might be most suitable for studies where different competing names are required. The remaining pairs showed various types of morphological relatedness. Most frequently, they consisted of a noun and a compound (28%; e.g. stoel-tuinstoel; *chair-garden chair*), or two compounds (12%; e.g kerstboom-denneboom; *christmas tree-pine tree*), or a noun and its diminutive form (7%; e.g. kaars-kaarsje; *candle*).

Concerning the semantic relation between the members of the pairs, we found that most commonly (46% of the pairs) they were (1) hypo-hypernym pairs, as in gitaar-elektrische gitaar (*guitar-electric guitar*), or aap-gorilla (*ape-gorilla*). Synonyms (2), broadly defined (as in wereldbol-globe; *globe*), occurred less often (31% of the pairs). Pairs could also be (3) closely related concepts (18%), e.g. salamander-hagedis (*salamander-lizard*); or (4) could refer to very different concepts (4%), e.g. koelbox-papierversnipperaar (*cooler-shredder*). These categories need to be validated, but give an initial impression of the kinds of names competing. Most useful for research might be (1) and (4).

Finally, although a cross-linguistic comparison was not the main goal of this work, we compared our results to those in the norming studies conducted by Brodeur and colleagues. Compared to the Canadian data set, Dutch modal NA (71%, sd = 23) was slightly higher (64% in Brodeur et al., 2010; 58% and 61% in Brodeur et al., 2014) and thus the average H-value (1.2, sd = 0.9) slightly lower (1.65 in Brodeur et al. 2010; 1.89 and 1.53 in Brodeur et al.2014). Thus, the Dutch participants used, on average, the modal name more frequently and used fewer alternative names per photograph than the Canadian participants. The object agreement scores in the two studies were similar (average 4.2, sd = 0.5 in the present study, compared to 3.90, sd = 0.50 in Brodeur et al. (2010) and 3.69, sd = 0.52 and 3.57, sd = 0.57 in Brodeur et al. (2014)). Correlations between variables (NA, H, and OA), were very similar too (see ***Table 3***).

**Table 3:** Correlations between the various dependent variables in the present study (Dutch) and the two studies of Brodeur et al. (2010, 2014).


	NAMING AGREEMENT			OBJECT AGREEMENT		

	DUTCH (*r*_*s*_)	2010 (*r*)	2014(*r*)	DUTCH (*r*_*s*_)	2010 (*r*)	2014 (*r*)

Object Agreement	.40**	.33*	.29*			

H-value	–.97**	–.96*	–.95*	–.45**	–.38*	–.35*


In sum, we present norms for written naming for 1397 photographs provided by young adult speakers of Dutch. The set includes 359 items with good name agreement, and it includes 35 items with two strong competing names. These sets might be most useful for designing studies. We highlight again that participants typed their responses. Spoken name agreement may deviate somewhat from the norms reported here because, for instance, some names are used more often in spoken than written language. Nonetheless, we hope that the current norms can assist researchers in their item selection.

## Data Accessibility Statement

All photographs, together with the norms (see Table B for all 1397 pictures, and Table C for norms for the useful set of 1185 pictures) and additional information on lexical competitors (Table D, D1, and D2) are available at *https://osf.io/kwu87/*.

In addition to the tables referred to in the manuscript, we uploaded a table (Table A1) with the raw data and a table (Table A2) with naming agreement for every used name for a certain picture after aggregating. Table A1 contains all individual responses (before aggregating; includes spelling mistakes and typo’s) and reports picture set (1–3), (anonymized) participant no., trial no., English picture name, used Dutch name, and object agreement score for each trial. This file will be of interest to researchers who want to try out different ways of summarizing the data, who are investigating the details of names given to pictures, or who are interested in spelling errors. Table A2 provides an overview of all different names used for each picture (after spelling check and aggregating), together with the proportion of participants that used this name (NA_all), calculated as the number of occurrences of this name (Used) divided by the number of participants that saw this picture (Nparts), i.e. including DKO and DKN respones. The OSF project also contains the R script used for all analyses (incl. all necessary input files; one of these files is a .txt file with the raw data (responses in column Input), note that in some trials there is an inconsistency between Button and Score. This is because some participants typed ‘a’ or ‘b’ instead of using the buttons. We removed the Input and manually changed the Score so these responses would be treated as a NO RESPONSE and could be categorized as DKO or DKN. However, we did not change the value in the Button column to keep the participant’s original response in the dataset.).
